# A mixed method study of an education intervention to reduce use of restraint and implement person-centered dementia care in nursing homes

**DOI:** 10.1186/s12912-017-0244-0

**Published:** 2017-09-18

**Authors:** Frode F. Jacobsen, Tone Elin Mekki, Oddvar Førland, Bjarte Folkestad, Øyvind Kirkevold, Randi Skår, Eva Marie Tveit, Christine Øye

**Affiliations:** 1 Center for Care Research, Western Norway, Bergen, Norway; 2 Western Norway University of Applied Sciences, P.O.Box 7030, N-5020 Bergen, Norway; 3 VID Specialized University, Bergen, Norway; 4 Uni Research Rokkan Centre, Bergen, Norway; 5 Norwegian University of Science and Technology (NTNU), Trondheim, Norway; 6 Norwegian National Advisory Unit on Ageing and Health, Oslo, Norway; 7 Western Norway University of Applied Sciences, Bergen, Norway; 8 Bergen Tourist Information, Bergen, Norway

**Keywords:** Education intervention, Implementation research, Nursing home staff, Person-centred care, Dementia care, Restraint, Leadership, Mixed methods

## Abstract

**Background:**

People living with dementia in nursing homes are most likely to be restrained. The primary aim of this mixed-method education intervention study was to investigate which factors hindered or facilitated staff awareness related to confidence building initiatives based on person-centred care, as an alternative to restraint in residents with dementia in nursing homes. The education intervention, consisting of a two-day seminar and monthly coaching sessions for six months, targeted nursing staff in 24 nursing homes in Western Norway. The present article reports on staff-related data from the study.

**Methods:**

We employed a mixed-method design combining quantitative and qualitative methods. The P-CAT (Person-centred Care Assessment Tool) and QPS-Nordic (The General Nordic questionnaire for psychological and social factors at work) instruments were used to measure staff effects in terms of person-centred care and perception of leadership. The qualitative data were collected through ethnographic fieldwork, qualitative interviews and analysis of 84 reflection notes from eight persons in the four teams who facilitated the intervention. The PARIHS (Promoting Action on Research Implementation in Health Services) theoretical framework informed the study design and the data analysis. Six nursing homes were selected for ethnographic study post-intervention.

**Results:**

Qualitative data indicated increased staff awareness related to using restraint - or not- in the context of person-centered care. A slight increase in P-CAT supported these findings. Thirteen percent of the P-CAT variation was explained by institutional belonging. Qualitative data indicated that whether shared decisions of alternative measures to restraint were applied was a function of dynamic interplay between facilitation and contextual elements. In this connection, the role of the nursing home leaders appeared to be a pivotal element promoting or hindering person-centered care. However, leadership-staff relations varied substantially across individual institutions, as did staff awareness related to restraint and person-centeredness.

**Conclusions:**

Leadership, in interplay with staff culture, turned out to be the most important factor hindering or promoting staff awareness related to confidence building initiatives, based on person-centered care. While quantitative data indicated variations across institutions and the extent of this variation, qualitative data offered insight into the local processes involved. A mixed method approach enabled understanding of dynamic contextual relationships.

**Trial registration:**

The trial is registered at Clinical Trials gov. reg. 2012/304 NCT01715506.

**Electronic supplementary material:**

The online version of this article (10.1186/s12912-017-0244-0) contains supplementary material, which is available to authorized users.

## Background

In this section, we first provide the general background with regard to dementia and restraint in Norwegian nursing homes (part 1.1), then briefly account for the overall intervention study (part 1.2), and lastly, provide a short introduction to the study reported in this article (part 1.3), of staff-related promoting and hindering factors for confidence building initiatives based on person-centred care to reduce restraint, which is the focus of this article reports.

### General background

Around 80% of people living in Norwegian nursing homes are cognitively impaired [1, 2]. Several studies have shown that people in nursing homes with cognitive impairment and high dependency are most likely to be restrained [3–5]. There is a need to reduce use of restraint in Norwegian nursing homes and to increase person-centered care and staff awareness of alternatives to restraint [6]. The concept of restraint is defined in different ways, but usually defined in relation to doing something against someone’s will and by that limiting free movement [7]. Use of restraint comprises: the use of physical restraint hindering freedom of movement (e.g. bedrails, belts, geriatric tables, lean back chairs or other physical devices hindering movement) [8], surveillance [9], relational restraint such as force in treatment (medical examination and hidden medication) or care situations [10, 11], and environmental restraint by isolating a resident in a locked room or locked facilities [12].

The Norwegian government has attended to the issue of use of restraint in somatic health care by introducing a new chapter in the Patient’s Rights Act in 2009 [13]. According to the law, the use of restraint in persons lacking the capacity to give informed consent should only be used as a last resort when ‘confidence building alternatives’ based on person-centred care have failed. The new legislation has been accompanied by a national education programme consisting of one-day seminars, information material and legal support from regional health authorities to support its implementation. Another nationwide program supported by the government is “The ABC of Dementia Care” [14]; a comprehensive workplace education program that encompasses knowledge of dementia and strategies to perform person-centred care. Despite these efforts, national surveys in 2011 and 2012 by the Health Authorities indicated that efforts at reducing restraints should be strengthened [6]. Neither of the government initiatives included coaching sessions for staff with regard to experienced challenging care situations in the nursing homes, which is a central part of the education intervention of our study.

This paper discusses quantitative and qualitative staff-related data embedded within a larger intervention study called MEDCED, described shortly in the section below. A combination of cluster- RCT, participatory action research (PAR) and ethnography was used to investigate patient and staff related effects, and the factors hindering or promoting the implementation across the nursing homes.

### The overall study: The MEDCED intervention study

The overall study was carried out among staff members in 24 nursing homes within the Western Norway Regional Health Authority, consisting of four Health Trusts. Six nursing homes were randomly selected for participation within four Health Trusts covering the Western region of Norway, totalling to 24 out of 83 eligible nursing homes. In homes with more than one ward, the nursing home director selected the ward to be included, with the exception of one nursing home, where two wards were included. After baseline sampling, the 24 nursing homes were randomized into an intervention group (12) and a control group (12). The sites were not stratified before randomization on the contextual characteristics to be dealt with later in the article. All staff members, including the ward leaders, all of whom being registered nurses (RNs), were invited to participate in the study. Staff employed with at least 50% full time equivalent position were included in the trial part of the study.

We chose a mixed method ‘intervention design’ ([15]: 44) combining a cluster-RCT with participatory action research (PAR) and ethnographic research because the intent was to evaluate both the effect of the intervention, and add qualitative data to study the influence of hindering and promoting implementation factors, as published elsewhere [16]. The study was informed by the Promoting Action on Research Implementation in Health Services (PARIHS) theoretical framework. The defined patient outcomes of the trial, restraint, agitation and use of psychotropic drugs, have been published elsewhere [10], and the study has been registered at Clinical Trials gov. reg.2012/304 NCT01715506. Table 1 details the different intervention activities in the MEDCED study and method approaches.

**Table 1:** Sequences of the overall MEDCED study

Phase	Pre –intervention 2011–2012	Per-intervention 2012–2013	Post-intervention 2013–2015
Intervention activities	Develop & prepare delivery of a standardized education content & methods; Workshops (4)	2 –days seminar +6 coaching sessions	Stakeholder meetings presenting & discussing findings (8)
Quantitative methods	Cluster- RCT; recruiting, randomization & single blinded baseline	Registration of fidelity issues according to WIDER recommendations & analyzing of baseline data	Cluster-RCT-7 months follow-up Descriptive statistics of restraint situation & fidelity issues Statistical analyses
Qualitative methods	Focus groups facilitators × 1–1,5 h × 3	Focus groups facilitators × 1,5 h × 1 Leader interviews (12) and registration of physical and geographic context elements Structured reflection notes informed by PARIHS context elements (86)	Ethnographic field studies (6 nursing homes/49 days), staff & leader interviews (42) Knowledge co-creation workshops research team & facilitators (5)
Mixed analyzing & knowledge construction			Analytical Meta-Interference, knowledge production & dissemination

The MEDCED education intervention was implemented by four twin teams with a total of eight persons, all of them registered nurses not part of our research team, with work experience from nursing homes, facilitating the intervention in 24 nursing homes over a period of 18 months, from September 2012 to May 2013. The facilitators received a 7 days course by members of the research team and also participated in several workshops as co-researchers, as published elsewhere [16]. In cooperation with the facilitators executing the education intervention, the education method and content of the written material were revised based on a previous intervention in four Norwegian nursing homes that had found promising results in terms of reduced use of restraint and psychotropic drugs in residents living with dementia [17]. During a 2 day seminar for all staff and their leaders, the facilitators presented a decision-making model ‘Trust before restraint’ (Tillit fremfor tvang, in Norwegian) that staff could use to make shared decisions of possible person-centred measures to avoid use of restraint in agitated residents living with dementia. In the seminars, they introduced the theoretical, ethical and legal foundation underlying the decision-making model, and, further the facilitators provided 1 hour coaching sessions each month for 6 months to assist the staff in applying the decision-making model related to specific and present challenging care situations identified by participating staff. The decision-making model offered a structured way for the staff to collectively address and agree to issues related to person-centred care [18], as well as the legal requirements. Further details of time, content and methods are published elsewhere [16, 19]. The nursing homes in the trial control group were offered the education intervention after 7 months follow-up ratings were collected (all 12 ‘control’ homes accepted the offer). Thus, we reiterated and up scaled the education intervention trial from four homes in a previous Norwegian research project by Testad et al. [17], to 24 nursing homes in the MEDCED study.

Findings from the trial data revealed unexpected low level of use of restraint in the nursing homes at the time of the baseline relative to previous national and international studies, measured as rate of patients subject to at least one means of restraint [10]. Despite the low baseline, however, a statistically significant reduction in use of restraint was found in all nursing homes during the intervention with a tendency to a greater reduction in the control group. Moreover, the patient population’s agitation score (CMAI) was significantly reduced, with a non-significant higher reduction in the control group. Changes in usage of psychotropic drugs were insignificant in both groups [10].

### Staff related factors reported in the MEDCED study

While the RCT data cannot offer any explanations to why the above mentioned findings resulted from the education intervention, the qualitative approach could provide possible explanations for the results by investigating intermediary factors, such as local forms of leadership and nursing home staff culture, dimensions not captured by quantitative data alone. Hence, the present article aim to provide, as a part of the overall study previously not published, insight into local leadership and staff cultures promoting or hindering person-centered care.

The findings discussed report from staff outcomes, based on the instruments The P-CAT (Person-centered Care Assessment Tool) and QPS-Nordic (The General Nordic questionnaire for psychological and social factors at work), and qualitative data related to person-centered care, all to be described below.

## How staff and their working contexts influence implementation of person-centered dementia care in nursing homes

### Theoretical framework and assumptions

We employed the PARIHS theoretical framework, which highlights the contextual elements of culture, leadership, and evaluation [20, 21], in other word, a broad range of contextual factors, among those staff related factors. Person-centeredness may be defined as “an approach to practice that is established through the formation and fostering of healthful relationships between all care providers, older people, and others significant to them in their lives. It is underpinned by values of respect for persons, individual right to self-determination, mutual respect, and understanding” [22:9].

Hence, the assumption is that increased person-centered knowledge and awareness makes it easier for the staff to find alternatives to use of restraint, since paying attention to the residents’ personality and habits may compensate for the residents’ lack of ability to express their wishes and needs. This lack of ability in verbally expressing themselves likely provokes agitation in the residents.

Education interventions are targeting individual’s motivation and learning skills. However, especially when the intervention aims to improve shared decision-making and concerted actions to put the knowledge into daily practice, as in the MEDCED intervention, it is also necessary to include a socio-cultural learning perspective. People learn as participators [22, 23] in social practices. Consequently, in order to explain success or failure of an intervention, the understanding of *why* and *how* contextual factors influence learning should be combinded with exploration of *why* and *how* particular contexts influence the staff’s motivation and skills to act according to their ‘new’ knowledge [24].

Contextual factors have proved to be important for reducing restraint in care work, as has been demonstrated for staff coverage and level of staff education [25]. One Swedish study concludes that the well-being of nursing staff is associated with the well-being of people with dementia in residential care settings [26] while another Swedish study demonstrates that implementing person-centered care improves staff wellbeing [27]. A Dutch national survey concludes likewise, adding the insight that implementation of person-centred care increases staff members’ experience of support by their leaders [28]. Hence, implementation studies paying attention to such contextual factors are highly warranted.

## Methods

The variables of interest were degree of person-centered care (as perceived by staff) and staff perception of leadership (see further details below). Building on experiences from the above mentioned project by Testad et al. [17], the questionnaires used to collect data on staff were the P-CAT form [29], measuring person-centred care, and the QPS-Nordic form [30], specifically focusing on the leadership dimensions of the form. A total of 349 staff members across the 24 nursing homes, staff members having at least 50% full time position, and of whom 299 (86%) responded to the staff survey at baseline and 228 (65%) at follow-up. P-CAT is an index created on the basis of responses from the care staff at the nursing homes. The respondents were given a list of 13 claims related to person-centred care at their institutions, to which they were asked whether they disagreed or not. A 5-point Likert-type scale was used for scoring purposes (ranging from 1 = “Disagree completely” to 5 = “Agree completely”). Adding these scores we created an index that could theoretically vary from 13 (minimum value and complete disagreement) to 65 (maximum value and complete agreement). QPS-Nordic is a general questionnaire for psychological and social factors in the work environment. A short form with a battery of 13 claims has been employed in the present research, focusing on the two dimensions of ‘control at work’ and ‘leadership’, the latter including seven leadership claims. These seven leadership claims were added together, constructing an index that could theoretically vary from seven (minimum value and complete disagreement that the nursing home’s management had an inclusive leadership style) to 35 (maximum values field and complete agreement that the nursing home’s management had an inclusive leadership style). In this study, we focus on P-CAT as our dependent variable, with QPS-Nordic employed as an independent variable.

Multistage focus group interviews with facilitators took place before (FG 1–3) and during (FG-4) the intervention. Focus groups were chosen instead of individual interviews, since the aim was to use the focus groups as forums for researchers reflecting together with the facilitators and develop insights together [19].

Six nursing homes selected for ethnographic fieldwork were chosen post-intervention with regard to maximum heterogeneity, as published elsewhere [16, 31]. The heterogeneity was related to contextual dimensions (staff and leadership characteristics, geographical and size characteristics) and diversity in leadership models, as well as high and low score on use of restraint and on agitation (CMAI) [32]. The heterogeneity sought was inspired by research literature pointing to the importance of contextual factors in facilitating and hindering education interventions in health care settings.

The ethnographic fieldwork was performed after the intervention, in six nursing homes in the intervention group. The fieldwork combined participant observation, and formal and informal interviews with staff, where observations contributed to formal and informal questions asked. The observations were made in living rooms, dining rooms, kitchens, gardens, halls and offices, lasting from five to 12 h a day for a total of 49 days, including observing 48 handover meetings during mornings, afternoons and evenings. Data consisted of field notes of observed actions and social interaction between staff members and between staff and residents or family. The fieldwork included formal interviews of a total of 28 nurses (nine were leaders), one social educator, one assistant occupational therapist, 19 nursing auxiliaries nurses and four assistant nurses.

With regard to leadership, the ‘directors’ refers to the top manager (in our sample they were all RNs), and the ‘leaders’ being the ones who were responsible for the care taking place in the participating wards in larger nursing homes. However, in the smallest nursing homes they only had one leader. Their titles also varied; nonetheless, for the purpose of our study we call them leaders because they participated in the study due to this part of their dual director and leadership role.

Findings in the quantitative dataset informed what to look for when analyzing the qualitative material, and, qualitative findings helped identify the important elements of a multilevel regression analysis, like the element of formal education. A comparative analytical multi-site fieldwork method [33] was used to analyze the ethnographic material illuminating the diversity in the implementation process.

The analysis of the qualitative data was conducted in three stages and performed by a team of four qualitative researchers. The first stage was a thematic and context specific thematic coding procedure [34], based on comparison of activities, conduct, perceptions, events and interaction in the different nursing home settings. The second stage was a focused coding procedure [34], based on comparison of activities, conduct, perceptions, events and interactions in the different nursing home settings. The third stage was informed by the PARIHS framework. This also pertains to reflection notes produced by the facilitators related to the coaching session, which were analyzed using direct thematic analyzing [16, 35], informed by the context sub-elements of ‘culture, leadership and evaluation’ in the PARIHS framework [19].

## Results

The results section reports on promoting and hindering factors for using confidence-building initiatives based on a person-centered approach as an alternative to restraint based on person-centered care, from qualitative and quantitative data. The quantitative and qualitative data provided different insights. The combined insights from quantitative and qualitative approaches were particularly important for analyzing staff characteristics and behavior.

### Staff and facility characteristics

The resident to staff ratio of the nursing homes averaged 2.9 residents per care staff member, measured as actual residents and actual staff on duty on a given day-shift on an ordinary weekday in June 2013. Regarding formal education background, there were 39.6% RNs, 54.9% auxiliary nurses (Licenced Practical Nurses, LPN/Licenced Vocational Nurses, LVN) and 5.4% care assistants. The level of education was significantly higher in the control group (see Additional file 1). When combining the results from baseline and follow-up we found that 46% of the staff in the control group reported having higher education, defined as university or university college level, whereas 34% of the staff in the intervention group reported having completed higher education.

An interview with the leaders after the follow-up measurements were completed revealed that all of the 24 nursing homes had participated in at least one government-initiated education program in dementia care, all running at the time of the onset of the education intervention in the present study. Those programs were related to the new legislation on use of restraint, and include the above mentioned ABC of Dementia Care [14] and the program linked to the introduction of the new legislation on use of restraint. The interviews with all 24 leaders revealed that 21 nursing homes out of 24 were actively involved in one or two of these education programs at the time of the intervention.

### Staff outcomes

The table below reports the mean score for all respondents (*N* = 452), with a total of 23 nursing homes (since responses from one nursing home are lacking in the follow-up), at the two different measurement points (baseline and follow-up) (Table 2).

**Table 2:** Mean P-CAT score at baseline and follow up

	N	Mean	SD	*P*-value^a^
Nursing homes, baseline	248	46.9	6,5	
Nursing homes, follow-up	204	47.8	6,9	
Diff: Follow-up vs. baseline (*p* value^a^)		0.9		0.078

^a^ *p* value for t-test (Pr(T < t))

The difference at the two different measurement points was not significant when distinguishing between control group and intervention group (Table 2).

As is seen from Table 3, both groups demonstrated a slight increase in the mean of P-CAT from baseline to the follow-up. These results are, however, aggregated means for the entire dataset with two groups. There was considerable variation between the different nursing homes.

**Table 3:** Mean P-CAT score at baseline and follow up, intervention and control group compared

		N	Mean	SD	*P*-value^a^
Intervention group	Nursing homes, baseline,	122	46.7	5.8	
	Nursing homes, follow-up	84	47.4	6.5	
	Diff: Follow-up vs. baseline (*p* value^a^)		0.8		0.192
Control group	Nursing homes, baseline,	127	47.0	7.2	
	Nursing homes, follow-up	120	48.0	7.2	
	Diff: Follow-up vs. baseline (*p* value^a^)		1.0		0.149

^a^ *p* value for t-test (Pr(T < t))

In Table 4 below, we report the result from a multilevel regression analysis, from the two different measurement periods (baseline and follow-up). Social background, work characteristics such as seniority, having used leadership responsibility and work hours as explanatory variables at individual level. Furthermore, we include QPS-Nordic is as explanatory variable. The nursing homes comprise the clustering groups (level 2).

**Table 4:** Multilevel regression analysis using P-CAT as dependent variable. Unstandardized coefficients with standard error in parenthesis. Data at Baseline and follow-up

	(1)	(2)	(3)	(4)
	Empty model baseline	Model w/explanatory variables, baseline	Empty model, follow-up	Model w/explanatory variables, follow-up
Age		−0.06 (0.04)		0.04 (0.04)
Gender (1 = female)		−1.67 (1.43)		0.01 (1.58)
Education (1 = higher education)		0.42 (0.90)		−0.63 (0.99)
Leader (1 = leader responsibility)		1.24 (1.31)		4.75 (1.69)
Senority (years)		0.09 (0.06)		−0.03 (0.07)
Week hours (work hours p/ week)		−0.10 (0.05)		0.02 (0.06)
QPS-Nordic		0.47 (0.08)		0.63 (0.08)
Constant	46.84 (0.63)	40.94 (3.43)	47.85 (0.72)	29.72 (3.81)
ICC	13.0	15.7	14.3	12.2
Observations	259	223	204	165
Observations	24	24	23	23

The intra-class correlation (ICC) indicates how much of the variation in the dependent variable can be explained by institutional belonging (nursing home). The ICC reveals that more than 13% of the variation in P-CAT is explained by institutional belonging. None of the explanatory variables were significant, except for week hours per week (baseline data) and having leader responsibility (follow-up data). The percentage of leaders in the intervention group (7.37%) responding at the follow-up is less than in the control group (13.94%) (Chi-quadrat test *p* = 0.147). We did not encounter a similar difference in the baseline measurement, where the percentage of leaders in the intervention group responding was 10.56% and 11.94% in the control group (Chi-quadrat test *p* = 0.717).

The ethnographic studies revealed a higher level of awareness in the staff after the intervention with regard to use of restraints. Post-intervention, the staff provided solid arguments for or against use of restraints in each and every situation evaluated, as one of the staff commented in an interview: “*One starts thinking, I have not thought that using a bedrail is use of restraint. You used to think it’s there to protect the resident*”. The facilitators’ role seemed important in improving the staffs’ level of awareness, as one staff member said: “*The facilitators were very inspiring; they made us open our eyes. It’s not like any ordinary course, because they made us stop for a bit and think things through*”. In addition, staff could appreciate the way they learned together by getting the opportunity to sit down for an hour and discuss resident cases, as one staff explained: “*Since we were all there sitting together we discussed and listened to each other. It is very important to listen to your colleagues’ experiences and learn from them, then you start thinking before you enter a resident room and then you are able to see the situation from the patients’ point of view*”. The staff achieved an increased awareness with regard to person-centered care, and more specifically, to a variety of confidence building measures as alternatives to use of restraints. However, as already pointed out and as shown in Table 4, the individual institutions varied with regard to agreement of whether confidence measures were perceived to be a positive change.

### Leadership as a promoting or hindering factor

In the second and fourth column of Table 4, we also included the QPS-Nordic variable. The QPS-Nordic instrument contains seven dimensions concerning the staff’s perception of their leaders. When treating P-CAT as the dependent variable and QPS-Nordic as the independent variable, we find a positive and significant correlation at both baseline and follow-up. Respondents who evaluated their leaders as open and inclusive were most likely to think that their institution is committed to person-centered care.

**Fig. 1:**
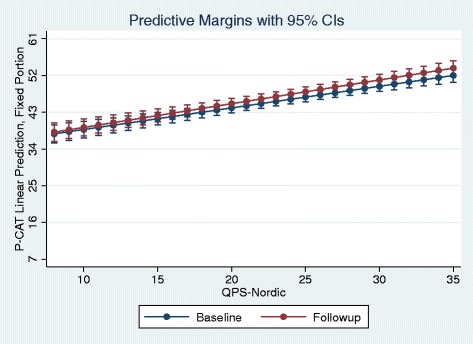
Adjusted predictions with 95% Cls

Below is a figure that predicts the likelihood of these positive and significant correlations at baseline and follow-up respectively: Fig. 1.

Respondents with a low QPS-N score (value 8) have a predicted P-CAT score around 38. P-CAT varies between 24 and 64, with an average of approximately 47 (see Table 3). For respondents with a maximum QPS-N score, with the value of 35 (hence evaluating their leaders highly positive), one finds a predicted P-CAT slightly below 53. All these predicted probabilities are controlled as to the effect of other variables.

The qualitative data pointed in the direction of large differences between the nursing homes as to leadership style and staff’s perception of their leaders. Moreover, how the leaders were involved or not in the implementation process varied considerably across the institutions. The ethnographic studies and the reflection notes from the facilitators, where also the number of participants were noted, both revealed that the leaders closest to the decision-making process played a pivotal role with regard to the potential success or failure of the intervention. The facilitators singled out leadership involvement as the most important factor for implementation. Their reflection notes revealed that the involvement of the leaders in the education intervention process influenced several dimensions of the decision-making process. Firstly, the mere presence of the leader turned out to increase the presence of the general staff at the supervision session. For instance we found in one nursing home that the number of staff attending supervision sessions declined from an average of 13 staff members in the first four sessions, to only five staff members for the remaining two when the leader was absent on sick leave. Secondly, the active involvement of the leader in the process enhanced the employment of agreed alternatives to restraint [36]. The reports from the facilitators and the ethnographic studies showed very different leadership practices. In one of the nursing homes, the leader was reported to be on leave for most of the intervention. In an interview, this leader initially voiced enthusiasm to participation in the intervention, but this enthusiasm did not seem to be communicated well to staff. This could be identified in limited commitment to the program among the general staff. In this site, few employees came to the supervision sessions, and the new knowledge was not embedded in the workplace culture, according to staff when interviewed. Yet, the staff at this institution had the highest level of education across the 24 sites.

In another nursing home, the staff experienced their leader as ‘distant’ and ‘lacking involvement in staff and resident matters’. Conversely, the ethnographic data revealed that this leader carefully mapped which residents shared things in common with each other and with staff members, and carefully planned for the ‘right matching’ and also, for the gradual implementation of the decision-making model. The leader gave freedom to staff with regard to how they organized their daily tasks, but she immediately intervened when the care work did not work out well [36].

## Discussion

Leadership and staff culture appear to be pivotal factors in promoting or hindering person-centered care, a necessary pre-condition for confidence building initiatives in staff-patient relationship, based on person-centered care. The combined insights from quantitative and qualitative approaches appeared particularly important for analyzing staff characteristics and behavior. Our research indicated a general strengthening of a person-centered approach in the participating nursing homes, at the same time as we discovered differences across the individual facilities.

The qualitative data indicated a development toward more person-centeredness compared to the situation before the education intervention. The quantitative data from P-CAT indicated a positive, but not a strong change in person-centeredness in the staff, with significance on the level of 10%. When reviewing the results from all the nursing homes at baseline and at the 7 months follow-up, the analysis point to a significant increase in agreement that the nursing home is providing more person-centered care.

The findings in the present article indicate an increased awareness in staff with regard to finding alternatives to use of restraint from baseline to follow-up, however, with a stronger support from qualitative than quantitative data. A slight increase (significant within 10%) in P-CAT in the staff was found in both the control and intervention group. Institutional belonging significantly predicted the variation in P-CAT and the variation in the seven leadership variables (claims) of the QPS-Nordic.The quantitative data (P-CAT) seems to some degree to support the qualitative findings that such an overall increase in awareness has been taking place. The collective learning process initiated by the education intervention, when staff collectively used the decision-making model to discuss especially challenging resident cases, may explain the increased level of awareness related to person-centred care. The employment of this decision-making model, under the continuous coaching of the facilitators, engaged the staff in a conscious and thorough decision process leading in each particular situation to either an alternative to restraints or a well-founded and explicitly justified use of restraints. However, this process seems to have had a somewhat paradoxical result of a lesser decrease in measured use of restraints in the intervention group, possibly because the staff grew more knowledgeable and were enabled to discriminate between measures falling within the juridical definitions of restraint [10]. The reflection notes from the facilitators and observations and interview data from fieldwork support the finding that such a process towards a higher critical awareness concerning person-centredness in care has taken place in most nursing homes, with some few exceptions [16, 32].

Important differences between the individual nursing homes appeared from the data analysis. The multilevel regression analysis on P-CAT baseline data indicated that institutional belonging is important with regard to staff perception of their leader. The same applies to the extent of person-centered care at their institution, lending support to the qualitative data identifying notable institutional differences [32]. This finding is in line with recent implementation research findings, which claim that contextual and institutional factors influence the success or failure of an intervention [37, 38]. Accordingly, local staff cultures evolve differently and act upon the education intervention in different ways, as shown in the qualitative part of the MEDCED intervention study [19, 32]. Findings in the multi-level regression analyses of P-CAT at baseline were nearly identical to the follow-up data. Hence, to provide results that are more robust, baseline data was chosen, since the N-value was higher than in the follow-up data.

The rich context and process data from the ethnographic studies and from the PAR study, which also gave numbers on attendance in the coaching sessions, provided valuable information as to promoting and hindering factors in implementation, where leadership stands out as a very important factor. As an example from the facilitator notes, in one home, the number of attendants dropped when the leader was on sick leave, from an average of 13 in the first four sessions, to five in the last 2 months when the leader was absent. Analyses of the reflection notes and ethnographic field notes showed that by acting as internal facilitators, the leaders’ activities directly and indirectly increased the potential for *success stories* in terms of more person-centered and restraint- free care to happen [16].

Leadership, moreover, turns out to be pivotal for increasing staff awareness and for implementing the decision-making model. This finding is in line with how recent organizational learning perspectives conclude on the importance of leadership for workplace learning processes [22, 23]. How staff perceived their leaders was found to predict how staff perceive presence or absence of person-centred care.

However, the qualitative data suggested that different leadership styles may promote as well as hinder the implementation of the decision-making model to find alternatives to use of restraint. Moreover, the quantitative data shown in Table 4 and Fig. 1 indicates that a positive staff evaluation of their leaders predicts a more positive perception of their institution as to commitment to person-centered care. International studies also support the assumption that leadership influences the outcome of education interventions [39–41]. The involvement of the leader led to an increased staff presence and active involvement in the supervision sessions and enhanced the use of the decision-making model in practice. However, the data from ethnographic fieldwork and the reflection notes from the facilitators demonstrate that leadership relates to knowledge implementation, and more generally, to staff motivation, in a complex way [16, 32, 36]. A complex relation between leadership and staff culture may explain the somewhat unexpected finding in one nursing home, which had the most positive results with regard to reduction in use of restraints: the leader was described by staff as ‘distant’ and disinterested in their daily work and workplace learning. In that particular nursing home, apart from the particular nature of the interaction between staff and leader, a staff culture characterized by collective learning and knowledge-sharing may have contributed to the positive result of the intervention [36].

Regarding possible connection between staff awareness and action, quantitative data from the trial seem inconclusive while qualitative data were more illuminating. Interviews and observations point towards a connection between raised staff awareness and increased attempts at finding alternative to restraint, and, a tendency to define more elements and situations as restraint after having gone through the education intervention; elements that the staff previously did not perceive as restraint [22].

## Conclusion

Within an education intervention targeting nursing home staff, this study aimed to investigate factors that hindered or promoted person-centred care, a pre-requisite to enhance confidence-building initiatives in dementia care. The staff-related qualitative data, and to some extent the staff-related quantitative data, indicated a development toward more person-centered care being performed compared to the situation before the education intervention. This finding may provide one possible explanation for reduced restraint and agitation found when analyzing the trial data related to patients in the overall study.

Involvement of leaders appeared to be a key issue in facilitating successful implementation. Both ethnographic studies and reflection notes systematically made during the implementation process by the facilitators substantiate this conclusion. The quantitative data lends support to this conclusion by indicating that a positive staff evaluation of leadership predicts for a positive appreciation as to the presence of person-centered care in the institution. The ethnographic studies make clear, however, that the manner in which the leaders are involved is important for the success or lack of success of the implementation.

The ethnographic data (based on participant observation and interviews), and data from reflection notes revealed a high level of awareness by staff about use of restraints. As a result of the training, the staff were able to provide more solid arguments for or against use of restraints in each and every situation they evaluated. They also did this in the context of promoting person-centered care.

Quantitative data to some extent supports this finding by indicating a moderate increase in P-CAT (significant within 10%), where P-CAT is a measure for the self-evaluation of the staff with regard to person-centeredness in care. Moreover, quantitative data indicate that both leadership, as perceived by staff, and person-centred care, vary significantly across the nursing homes. The importance of the context of individual nursing homes is indicated by both quantitative and qualitative findings.

### Methodological limitations and strengths

Even though the selection of nursing homes was completely random, the baseline data revealed differences in a number of factors between the intervention and control groups. The higher percentage of leaders and the higher level of formal education in the control group are other selection biases that may have influenced the results. The lack of an identifying marker making it possible to follow the individual respondents from the baseline to the follow-up measurements is a possible weakness of the study. Nevertheless, given that the participating wards are small and a relatively high average length of service of the staff, it is likely that there is a substantial overlap between the respondents of the first and second round of measurements. Lastly, the qualitative data in the study are mainly from nursing homes in the intervention group.

This study has benefitted from a mixed method approach. Qualitative data employed in tandem with quantitative data, as in our study, have the potential to increase the contextual understanding and offer possible causal explanations for *how* the implementation develops, and under *what* circumstances. That is, these approaches help identify promoting and hindering factors in complex interplay.

## Additional file

Additional file 1: Overview of independent variables, number of respondents (N) and mean with standard deviation in parenthesis. Control and intervention group compared at baseline and follow-up. (PDF 172 kb)
