# Supplementary material for: A mixed method study of an education intervention to reduce use of restraint and implement person-centered dementia care in nursing homes
Source: BMC Nurs. 2017 Sep 18;16:55. doi: 10.1186/s12912-017-0244-0 (PMC5604397; doi:10.1186/s12912-017-0244-0)
Supplement: Additional file 1: — Overview of independent variables, number of respondents (N) and mean with standard deviation in parenthesis. Control and intervention group compared at baseline and follow-up. (PDF 172 kb) [file 12912_2017_244_MOESM1_ESM.pdf]

Additional file 1: Overview of independent variables, number of respondents (N) and mean with standard deviation in parenthesis. Control and intervention group compared at baseline and follow-up.

	Control group				Intervention group			
	**Baseline**		**Follow-up**		**Baseline**		**Follow-up**	
	N	Mean	N	Mean	N	Mean	N	Mean
Age	129	46.20 (12.53)	120	46.32(12.68)	143	45.73 (12.71)	96	45.30 (12.30)
Gender (1=female)	129	1.95 (0.21)	121	1.96 (0.20)	139	1.90 (0.30)	98	1.88 (0.33)
Education (1=higher education)	131	0.48 (0.50)	125	0.43 (0.50)	144	0.32 (0.47)	97	0.37 (0.49)
Leader (1=leader responsibility	134	0.12 (0.33)	126	0.13 (0.34)	142	0.11 (0.31)	95	0.07 (0.26)
Senority (years)	134	8.17(8.05)	124	9.08 (8.40)	140	8.72 (9.30)	95	8.71 (8.61)
Week hours (work hours p/ week)	127	29.03(8.26)	122	30.79 (7.32)	137	28.98 (8.35)	92	29.07 (7.58)
QPS-Nordic	132	25.36(5.09)	123	25.24 (5.69)	140	25.46 (4.71)	96	24.10 (5.45)
